# Supplementary material for: Recurring seasonality exposes dominant species and niche partitioning strategies of open ocean picoeukaryotic algae
Source: Commun Earth Environ. 2024 May 20;5(1):266. doi: 10.1038/s43247-024-01395-7 (PMC11106004; doi:10.1038/s43247-024-01395-7)
Supplement: Supplementary file 2 — Supplementary Information [file 43247_2024_1395_MOESM2_ESM.pdf]

**Supplementary Information for “Recurring seasonality exposes dominant species and niche partitioning strategies of open ocean picoeukaryotic algae”**

Charlotte A. Eckmann[1,2], Charles Bachy[3,4], Fabian Wittmers[1,3], Jan Strauss[3], Leocadio Blanco-Bercial[5], Kevin L. Vergin[6], Rachel J. Parsons[5], Raphael M. Kudela[1], Rod Johnson[5], Luis M. Bolanos[7], Stephen J. Giovannoni[7], Craig A. Carlson[8] & Alexandra Z. Worden[1,2,*]

[1]Marine Biological Laboratory, 7 Mbl St., Woods Hole, MA 02543, USA, [2]Ocean Sciences Department, University of California Santa Cruz, Santa Cruz, CA 95064, USA, [3]Ocean EcoSystems Biology Research Unit, GEOMAR Helmholtz Centre for Ocean Research Kiel (GEOMAR), Kiel, DE, [4]Sorbonne Université, CNRS, FR2424, Station Biologique de Roscoff, Roscoff 29680, FR, [5]Bermuda Institute of Ocean Sciences—Arizona State University, 25 Biological Station, St. George's, Bermuda, [6]Microbial DNA Analytics, Phoenix, OR 97535, USA, [7]Department of Microbiology, Oregon State University, Corvallis, OR 97331, USA, [8]Department of Ecology, Evolution, and Marine Biology, Marine Science Institute, University of California Santa Barbara, Santa Barbara, CA 93106, USA

*Corresponding author: azworden@mbl.edu, MBL, 7 MBL Street, Woods Hole, MA 02543

**Table of Contents**

Supplementary Note 1……………………………………………………………..............................3

Fig. S1. Biogeochemical variability at the BATS site ……………………………………...5

Fig. S2. 16S rRNA gene tree reference tree ………………………………………………...6

Fig. S3. Comparison of Micromonas ASVs ………………………………………………...8

Fig. S4. Identity Matrix for putative Clade IX sequences …………………………….......10

Fig. S5. Prasinophyte contributions by depth layer and stability period ……………….....11

Fig. S6. Distribution of prasinophyte ASVs...................………………………………….12

Fig. S7. Partial canonical correspondence analysis................................…………….........13

Fig. S8. Rarefaction analysis......................……………………………….....……......14

Fig. S9. Rarefaction analysis plastids...................……………………………......……......15

Supplementary References………………………….................…………………..16

**Supplementary Note 1**

Some prasinophytes demonstrated strong partitioning connected to stability periods and depth, while others were persistently present (Fig. 6a,b, Table S1). Persistent sub-species variants exhibited greater overlap between years (Fig. 6c) and included taxa with differencing presence levels (some at low and some at high relative abundances). For example, in 2017 putative Class IX ASV3858 (average 1.2±1.6% of plastid amplicons across samples where detected) was present in all stability periods with its largest relative contributions (7.1%) in the AT between 40-80 m (Fig. 6b). Other persistent ASVs were Class II sub-species variants with the highest relative abundances among prasinophytes—specifically, *Ostreococcus* Clade OII ASV77 and *B. calidus* ASV177 (Fig. 6, Fig. 4). These ASVs were abundant throughout the euphotic zone during DM and ST and then present at the DCM during SS and AT. *Ostreococcus* Clade OII ASV6 exhibited persistence in 2017 and was detected in all but the AT in 2018 and 2019. *M. commoda ss* ASV61 was detected in three stability periods at multiple depths (all but AT) in 2017 and 2018, and was persistent in 2019, similar to *Micromonas* candidate species 1 ASV81 which was generally detected in all stability periods except AT. Some persistent ASVs—such as ASV3858 and putative IX ASV1638—were present at the SS surface and DCM, while others—such as ASV77 and ASV81—were found only below the SS ML, mostly at the DCM.

The stability period with the highest number of ASVs in the euphotic zone (74 in total from 140 m and above) was the SS (Fig. 6a,b). Forty-three of these were exclusive to the SS, the largest proportion of ephemeral ASVs unique to any stability period. Three SS unique ephemerals were detected across all years: Class I ASV5929, *Ostreococcus* OII ASV33032, and Class VI *Prasinococcus* ASV17508 (Fig. 6d). These three ASVs— along with three undetermined *Micromonas* A/B-lineage ASVs, two Class IV, another Class VI, and putative Class IX—were the most relatively abundant of the summer exclusive ASVs (each contributing at least >1% of plastid amplicons in one or more samples). The most abundant one-stability-period-exclusive ephemerals for AT and DM belonged to Class II; in spring, none contributed over 1% of plastid amplicons but Class II still had the highest-contributing ASVs. Thus, exclusively SS ephemerals exhibited considerable interannual variation (Fig. 6d).

Of the 74 prasinophyte ASVs detected during the SS (but not necessarily exclusive to the SS), 17 were detected in all three years, 9 appeared only in 2018 and 2019, and two only in 2017 and 2019 (Fig. 6a, Fig. S6c). The distributions of the 17 SS 'all years' ASVs differed with depth. In

the SS upper 5 m the summed mean relative plastid contributions of the ‘all years’ ASVs detected (8 in total) was 2.2±2.4%, with *M. commoda ss* ASV273 and putative Class IX ASV3019 and ASV1248 as dominants (Fig. S6b). At 40 m the community had shifted such that the ‘all years’ set consisted of 13 ASVs with a mean of 3.3±2.5% of plastid amplicons with putative Class IX ASV3019 and Class I ASV3768 dominating prasinophytes. Contributions from Class VI *Prasinococcus*, Class II *Ostreococcus* Clade OII, *M. commoda ss*, and *M. bravo* were lesser but still notable. Most SS ‘all years’ ASVs (16 of 17) were found at the DCM (80-120 m); collectively, these ranged to 71.5% of plastid relative abundances, with *Ostreococcus* Clade OII being dominant, while *B. calidus*, *Micromonas* (*commoda ss*, *bravo*, and candidate species 1), and putative IX had lesser contributions (Fig. S6b). Three ‘all years’ ASVs (*Ostreococcus* OII ASV2949 and ASV33032, *M.* candidate species 1 ASV81) were found only at or below the DCM.

Ephemeral SS sub-species variants also exhibited depth variations. *M.* candidate species 2 ASV1156 formed up to 8.6% of plastid amplicons in the 2019 SS surface but was not detected below 40 m or in other years (Fig. S6b, S7b). A similar trend was observed among other non-dominant ASVs in other stability periods, such that several contrasted with distributional patterns of the dominant ASVs from that species (Table S1). Moreover, some ephemeral sub-species variants appeared at depths where the dominant variant was rare or not detected (e.g., Fig. S6c-d). For other groups, seasonal trends were more consistent across ASVs; for example, Class I and Class VI *Prasinococcus* ASVs were mostly found during the SS (Figure S6a,e), together comprising 0.57±2.4% of SS plastid amplicons.

## Supplementary Figures

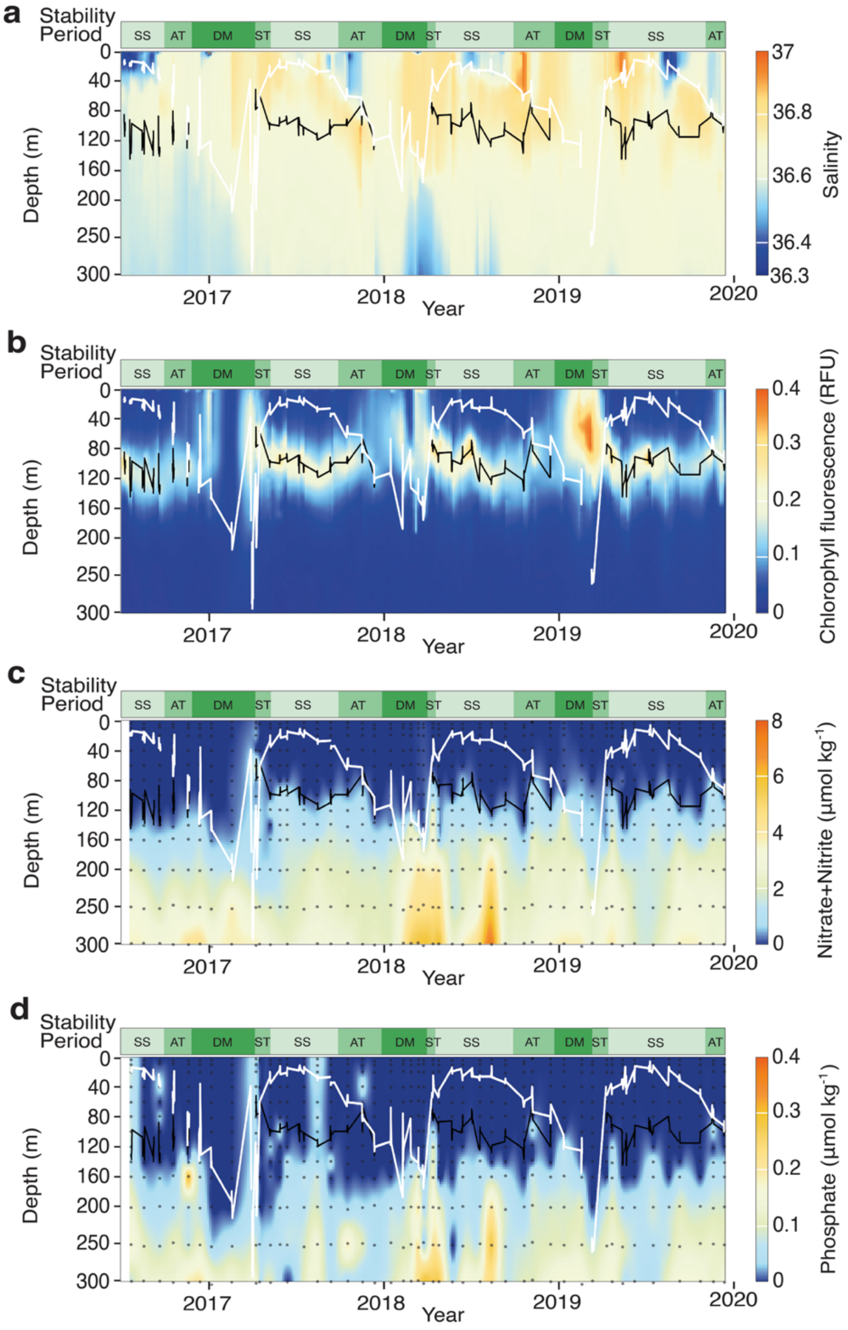


**Fig. S1. Biogeochemical variability at the Bermuda Atlantic Time-Series Study (BATS) site between July 2016 and December 2019.** (a) salinity, (b) CTD-sensor derived chlorophyll fluorescence, (c) nitrate + nitrate (µmol $kg^{-1}$) and (d) phosphate (µmol $kg^{-1}$) determined using spectrophotometric approaches and plotted based on interpolation from discrete data points (black dots corresponding to 13 depths per profile). Superimposed are lines indicating the DCM (black) and MLD (white). The stability periods (SS= stratified summer, AT= autumn transition DM= deep mixing, and ST= spring transition) are indicated by a green bar and are shown as such in other relevant figures.

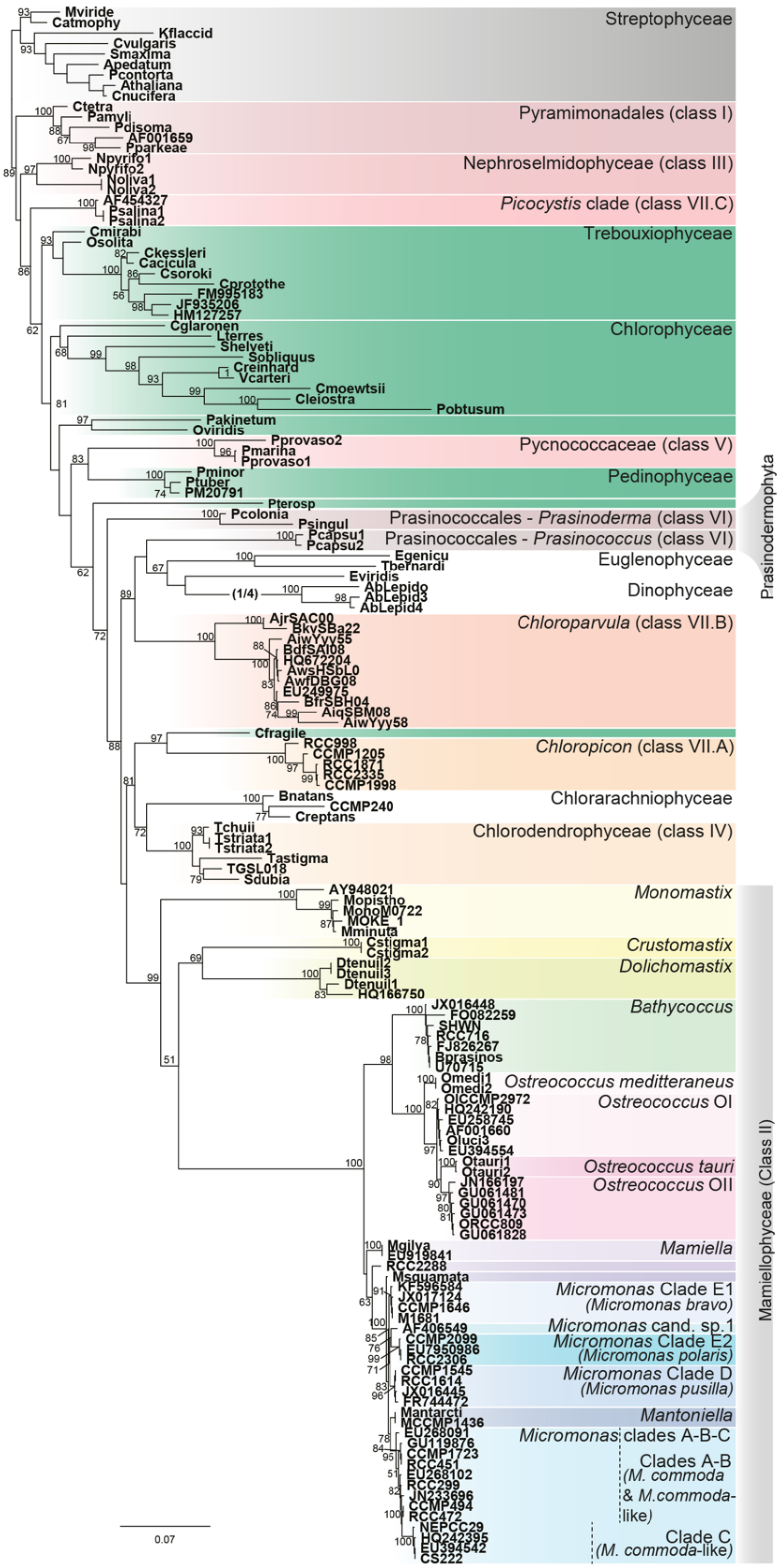


**Fig. S2. 16S rRNA gene tree reference tree for placement of Viridiplantae amplicons.** The reconstruction consists of 149 near full-length sequences with nine streptophyte sequences used as outgroup. Bootstrap values are indicated by the number at the node. Prasinophyte groups (as

well as Prasinodermaphyta, i.e., former prasinophyte Class VI) are designated by a variety of color gradients, with other Viridiplantae groups in dark green and non-Viridiplantae groups from secondary endosymbiosis with no color gradient.

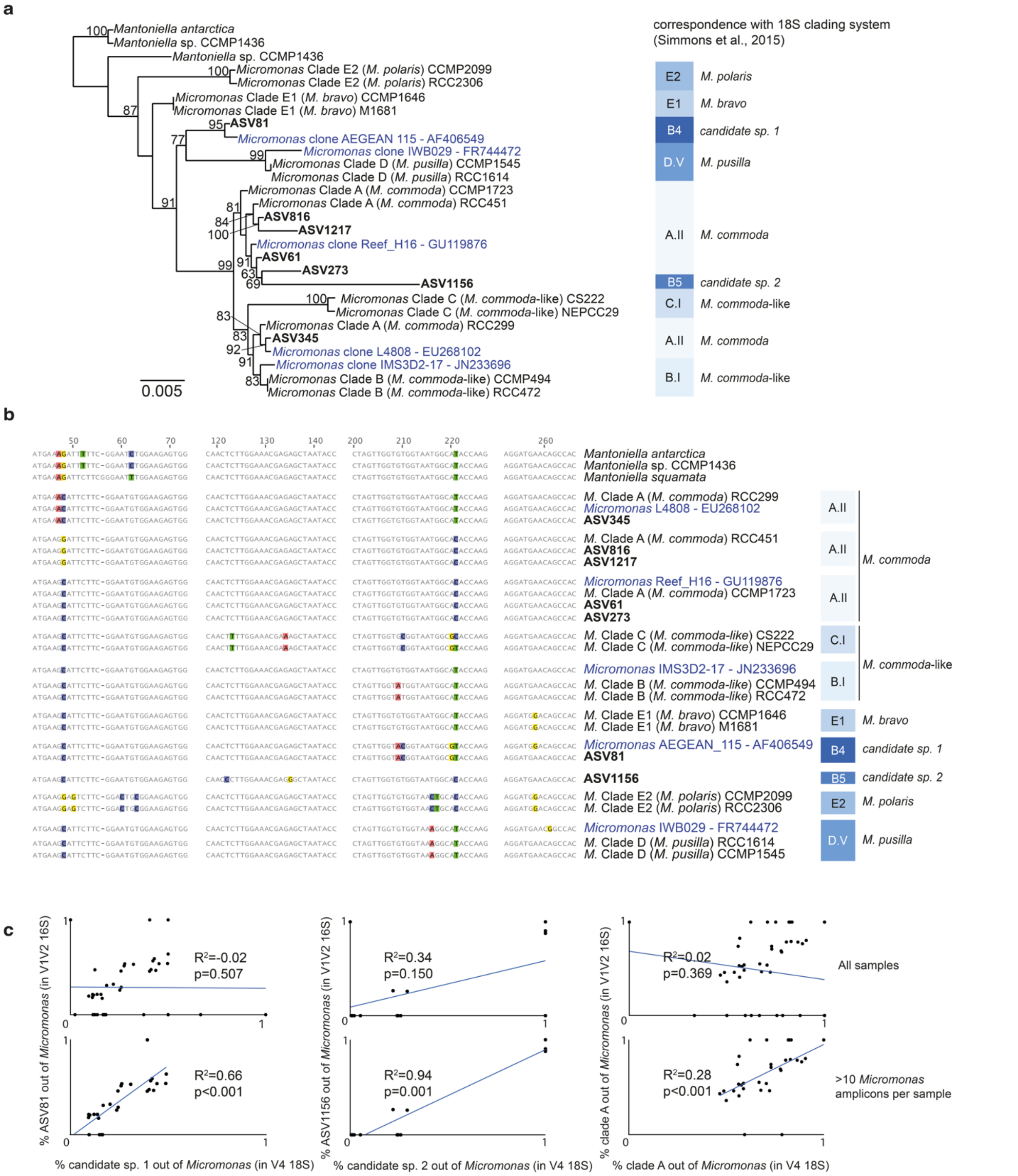


**Fig. S3. Comparison of *Micromonas* ASVs from this study to known *Micromonas* sequences from culture and environmental isolates.** (a) Maximum Likelihood phylogenetic tree of V1-V2 16S rRNA sequences of *Micromonas* (all sequences trimmed to V1-V2 16S amplicon size). Environmental sequence names are in blue font, sequences from culture in black font, and *Micromonas* ASVs from this study indicated in bold black font. Corresponding groups were

labelled according to naming determined with near full-length 18S rRNA sequences by [1] (b) comparison of alignments of the 16S gene V1-V2 region of *Micromonas* sequences in the above tree. (c) linear regression comparing percent contribution of 16S ASV81 to *Micromonas* amplicons in 16S to percent contribution of *Micromonas* candidate species 1 to *Micromonas* amplicons in 18S for all samples with either assignment (n=43), then samples with greater than 10 *Micromonas* amplicons (n=30). The same was done with ASV1156 and *Micromonas* candidate species 2 (n=16 and 14, respectively) and *M. commoda* clade A (n=73 and 33, respectively). The adjusted coefficient of determination ($R^2$) values from the linear regression and p-values from the Spearman correlations are shown.

| | ASV22206 | ASV41934 | ASV57981 | ASV35374 | ASV1638 | ASV17799 | ASV3858 | ASV7021 | ASV6637 | DQ438491 | ASV26923 | ASV1248 | KX938210.1 | ASV3019 | MG488520.1 | ASV2562 | KX938212.1 | ASV30087 | ASV63408 | JX945378.1 |
|---|---|---|---|---|---|---|---|---|---|---|---|---|---|---|---|---|---|---|---|---|
| ASV22206 | 100 | 98 | 98 | 98 | 99 | 99 | 88 | 87 | 88 | NA | 92 | 89 | 90 | 91 | 91 | 91 | 91 | 89 | 88 | 90 |
| ASV41934 | 98 | 100 | 99 | 100 | 99 | 99 | 89 | 88 | 89 | NA | 93 | 90 | 90 | 91 | 91 | 91 | 92 | 88 | 88 | 91 |
| ASV57981 | 98 | 99 | 100 | 99 | 100 | 99 | 89 | 89 | 89 | NA | 93 | 90 | 91 | 92 | 92 | 92 | 92 | 88 | 88 | 91 |
| ASV35374 | 98 | 100 | 99 | 100 | 100 | 99 | 89 | 88 | 89 | NA | 93 | 90 | 91 | 92 | 92 | 92 | 92 | 88 | 88 | 91 |
| ASV1638 | 99 | 99 | 100 | 100 | 100 | 100 | 89 | 89 | 89 | NA | 94 | 91 | 91 | 92 | 92 | 92 | 92 | 89 | 88 | 91 |
| ASV17799 | 99 | 99 | 99 | 99 | 100 | 100 | 89 | 88 | 89 | NA | 93 | 90 | 91 | 92 | 92 | 92 | 92 | 88 | 88 | 91 |
| ASV3858 | 88 | 89 | 89 | 89 | 89 | 89 | 100 | 90 | 92 | NA | 93 | 92 | 92 | 92 | 92 | 92 | 92 | 89 | 91 | 94 |
| ASV7021 | 87 | 88 | 89 | 88 | 89 | 88 | 90 | 100 | 97 | NA | 92 | 93 | 93 | 93 | 93 | 93 | 93 | 89 | 89 | 92 |
| ASV6637 | 88 | 89 | 89 | 89 | 89 | 89 | 92 | 97 | 100 | NA | 94 | 94 | 94 | 94 | 94 | 94 | 94 | 90 | 90 | 93 |
| DQ438491 | NA | NA | NA | NA | NA | NA | NA | NA | NA | 100 | NA | NA | 95 | NA | 48 | NA | 94 | NA | NA | 89 |
| ASV26923 | 92 | 93 | 93 | 93 | 94 | 93 | 93 | 92 | 94 | NA | 100 | 95 | 95 | 97 | 97 | 97 | 97 | 93 | 94 | 96 |
| ASV1248 | 89 | 90 | 90 | 90 | 91 | 90 | 92 | 93 | 94 | NA | 95 | 100 | 100 | 98 | 98 | 97 | 98 | 95 | 92 | 95 |
| KX938210.1 | 90 | 90 | 91 | 91 | 91 | 91 | 92 | 93 | 94 | 95 | 95 | 100 | 100 | 99 | 98 | 98 | 99 | 95 | 92 | 94 |
| ASV3019 | 91 | 91 | 92 | 92 | 92 | 92 | 92 | 93 | 94 | NA | 97 | 98 | 99 | 100 | 100 | 99 | 100 | 94 | 92 | 95 |
| MG488520.1 | 91 | 91 | 92 | 92 | 92 | 92 | 92 | 93 | 94 | 48 | 97 | 98 | 98 | 100 | 100 | 99 | 99 | 94 | 92 | 95 |
| ASV2562 | 91 | 91 | 92 | 92 | 92 | 92 | 92 | 93 | 94 | NA | 97 | 97 | 98 | 99 | 99 | 100 | 100 | 94 | 92 | 95 |
| KX938212.1 | 91 | 92 | 92 | 92 | 92 | 92 | 92 | 93 | 94 | 94 | 97 | 98 | 99 | 100 | 99 | 100 | 100 | 94 | 92 | 94 |
| ASV30087 | 89 | 88 | 88 | 88 | 89 | 88 | 89 | 89 | 90 | NA | 93 | 95 | 95 | 94 | 94 | 94 | 94 | 100 | 96 | 93 |
| ASV63408 | 88 | 88 | 88 | 88 | 88 | 88 | 91 | 89 | 90 | NA | 94 | 92 | 92 | 92 | 92 | 92 | 92 | 96 | 100 | 97 |
| JX945378.1 | 90 | 91 | 91 | 91 | 91 | 91 | 94 | 92 | 93 | 89 | 96 | 95 | 94 | 95 | 95 | 95 | 94 | 93 | 97 | 100 |

% Identity
100
95-99
90-94
85-89

**Fig S4. Identity Matrix for putative Clade IX sequences.** The goal of the matrix was to determine whether 15 low relative abundance ASVs sequenced at BATS could connected to known prasinophyte 18S rRNA genes. These sequences were mostly present in the SS, particularly ASV2562 and ASV3019 (99% identity to each other). With respect to prior discoveries, these 15 related sequences were most similar (91-99% identity) to an uncultivated group from the North Pacific subtropical gyre [2]. These uncultivated groups from the North Pacific shared ~98% identity with an East China Sea sequence (DQ438491) that is linked in $PR^2$ to putative prasinophyte Class IX 18S rRNA sequences, although the basis for this linkage is unclear (this sequence lacks the start of the 16S gene, i.e., the V1-V2 region, as do many $PR^2$/Phytoref sequences). We did not find a statistical link between 'putative Class IX' and 18S rRNA Class IX as assigned by $PR^2$ in our amplicon data; however, relative amplicons abundances from different markers are subject to different biases.

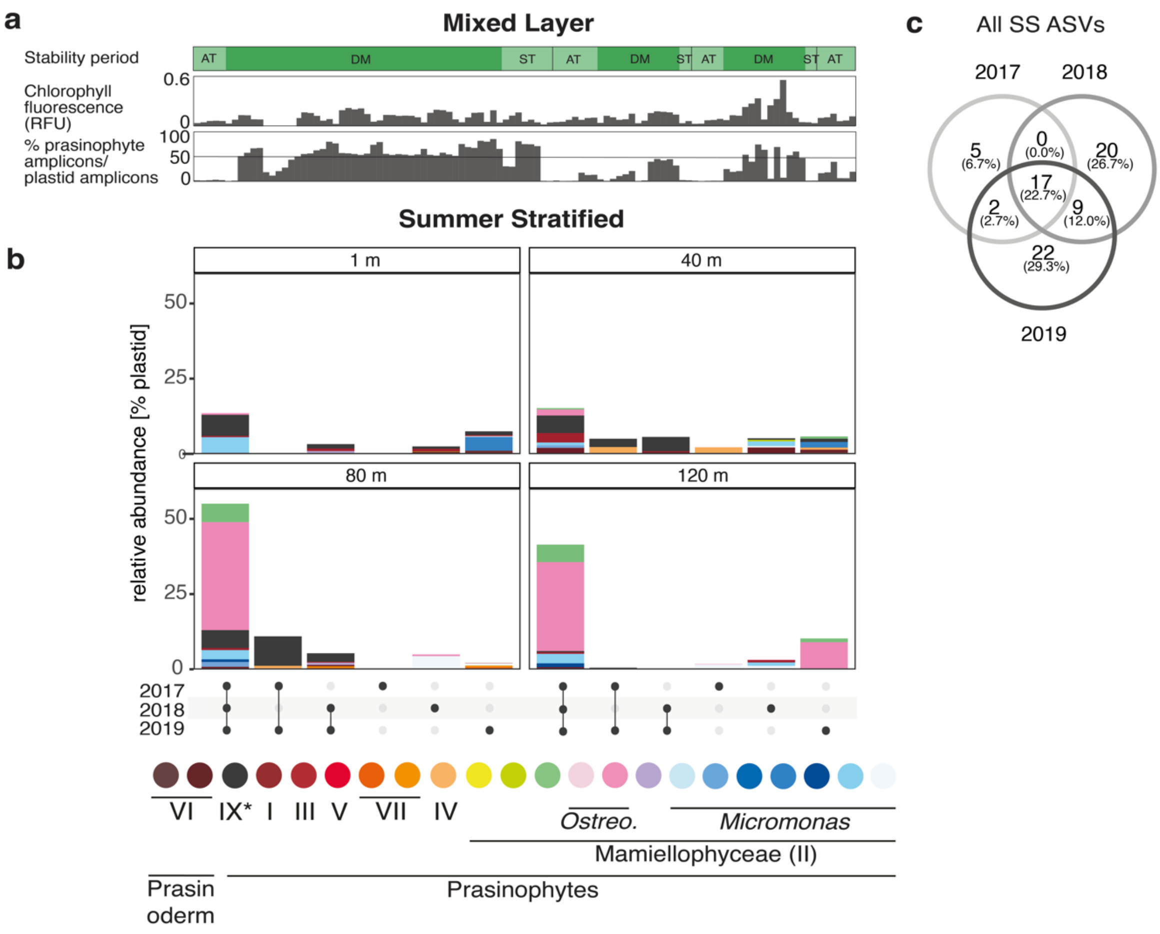


**Fig. S5. Prasinophyte contributions by depth layer and stability period.** (a) Percent prasinophyte amplicons out of plastid amplicons in the mixed layer with a line indicating the 50% contribution level. Note the X-axis represents sampling dates and is not scaled linearly according to time, due to heavier sampling during highly dynamic periods. Months with an asteria indicate where sampling did not take place at those depths. Water column stability l (SS= stratified summer, AT= autumn transition, DM= deep mixing, and ST= spring transition) are indicated by the green bar. (b) Bar plots of average relative plastid abundances of prasinophyte ASVs detected during the SS, with each quadrant a separate depth and the x-axes delineating in which years they were detected. Even when considering just the surface 1 m, 2018 and 2019 still had the greatest number of SS-exclusive ASVs (c) Venn diagram of all ASVs detected in the SS mixing period but not necessarily exclusive to that mixing period.

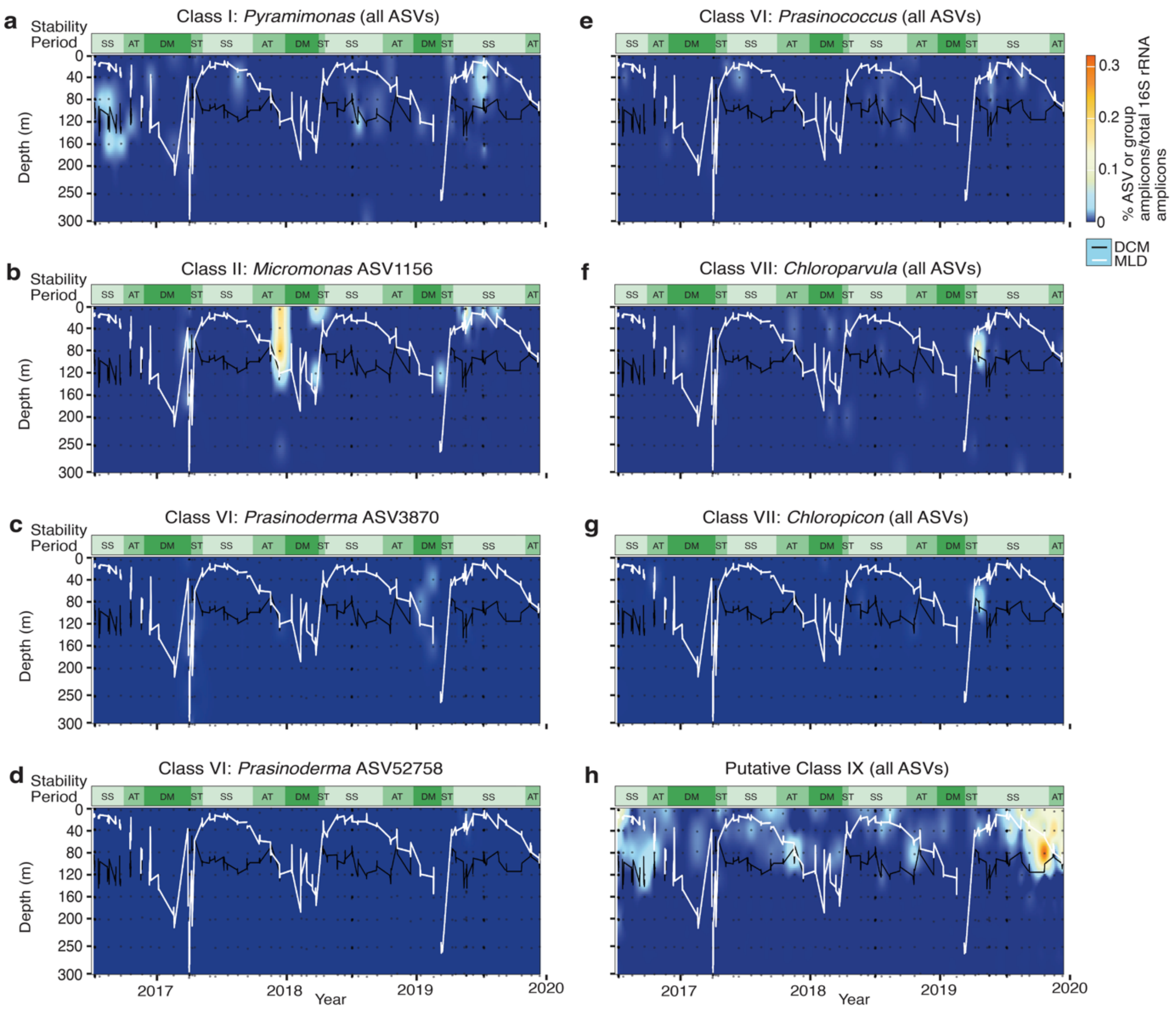


**Fig. S6. Distribution of prasinophyte ASVs.** Relative abundances of (a) Class I *Pyramimonas* (all ASVs), (b) Class II *Micromonas* candidate species 2 ASV 1156, (c) Class VI *Prasinoderma* ASV 3870, (d) Class VI *Prasinoderma* ASV52758, (e) VI *Prasinococcus* (all ASVs), (f) Class VII *Chloroparvula* (all ASVs), (g) Class VII *Chloropicon* (all ASVs), (h) putative Class IX (all ASVs) out of total amplicons at a range of depths from 1 m to 300 m from July 2016 to December 2019 based on interpolation from discrete data points (black dots corresponding to the 8 depths per profile). These data illustrate that some of the more common ASVs are not detected at depths where ephemerals appear. For instance, Class IV *Prasinoderma* ASV3870 made up 77.4% of all *Prasinoderma* amplicons and was found primarily at DM and ST at a median depth of 120 m, while ASV52758 made up 16.8% and was found only at SS in the upper euphotic zone (panels c and d). Note that all except for (b) represent a sum of all ASVs for that group. The stability periods, defined by DCM and MLD, are indicated by a green bar.

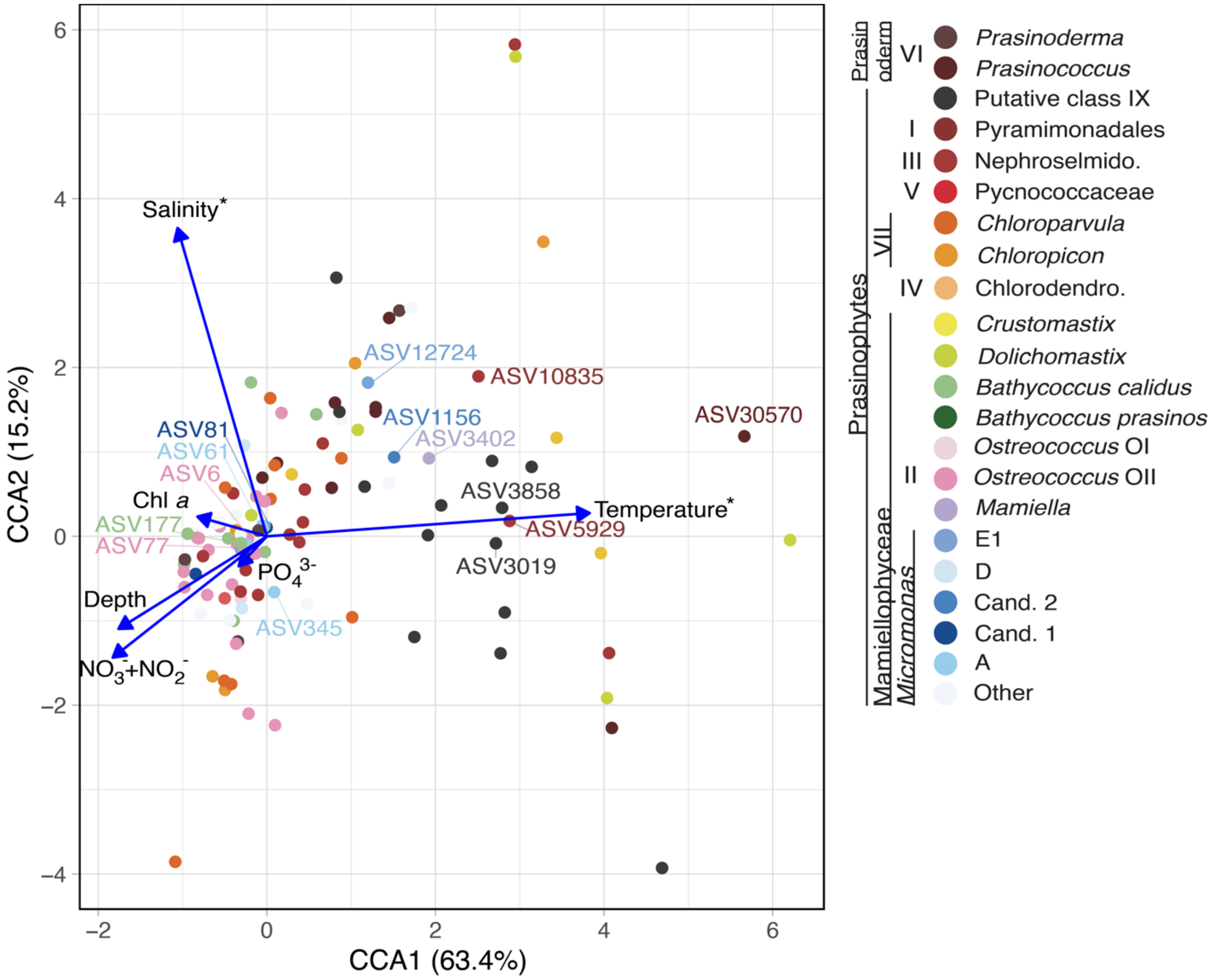


**Fig. S7. Relationship of prasinophyte ASVs to environmental variables.** Partial canonical correspondence analysis plot of prasinophyte ASVs with water column stability period as the conditioning variable, with vectors representing temperature (ºC), salinity, nitrate + nitrite (µmol kg$^{-1}$), phosphate (µmol kg$^{-1}$), and chlorophyll *a* (µg kg$^{-1}$). The proportion of variation (inertia) explained by the constrained eigenvalues is indicated in parentheses for each axis. Vectors with an asterix indicate that variable was significantly correlated with the variation in the prasinophyte ASVs. Prasinophyte groups are indicated by color, with several important ASVs labelled by number.

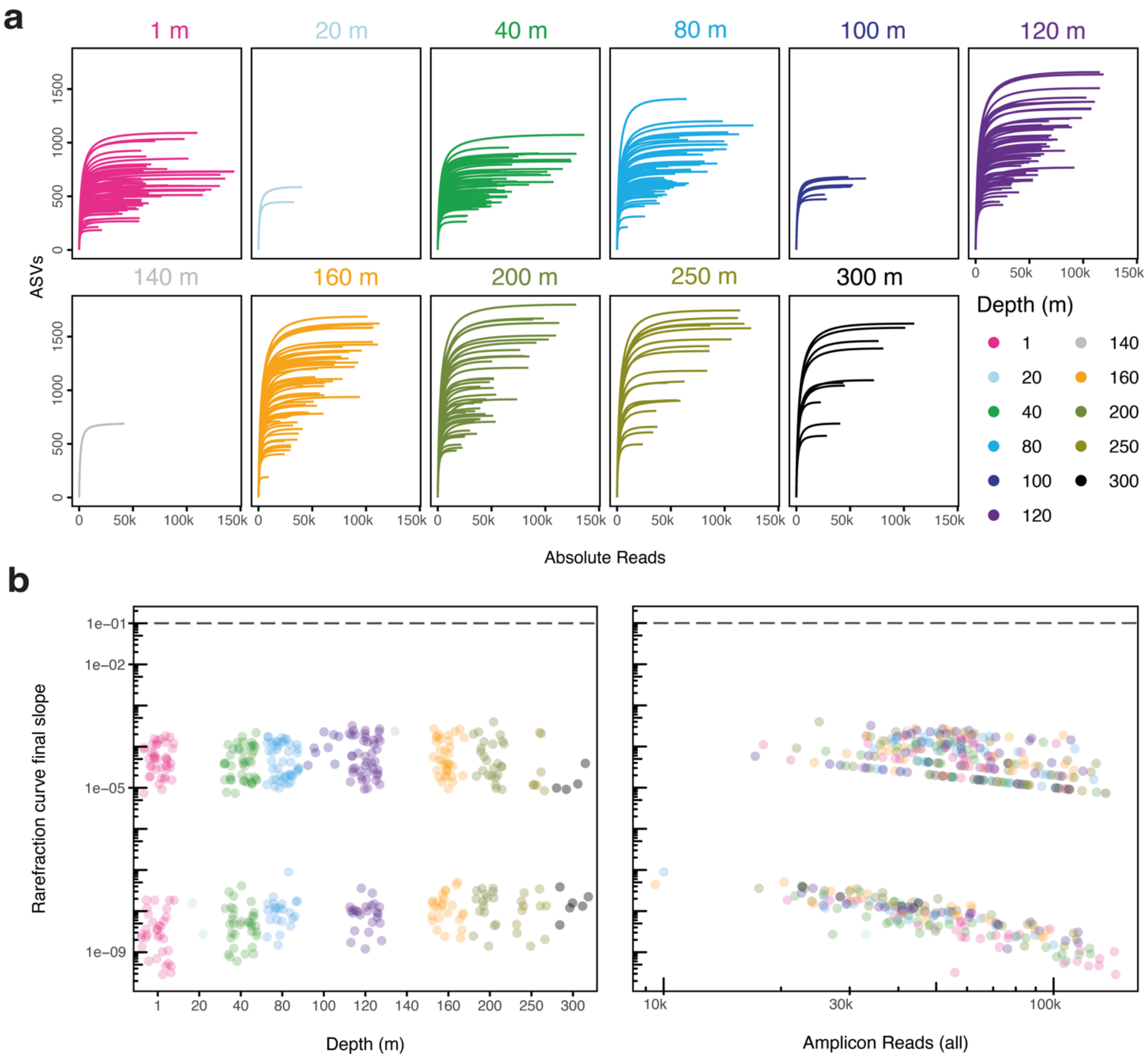


**Fig. S8. Rarefaction analysis for the 431 samples used in the study** at all depths down to 300 m to determine sequencing saturation (samples from 1-140 m with <50 plastid amplicons were not considered). (a) Rarefaction curves with number of ASVs vs. number of absolute reads. (b) The final slope of the rarefaction curves plotted vs. depth and number of amplicons. Final slopes of below 0.1 are plotted below the dotted line and are considered to have reached saturation.

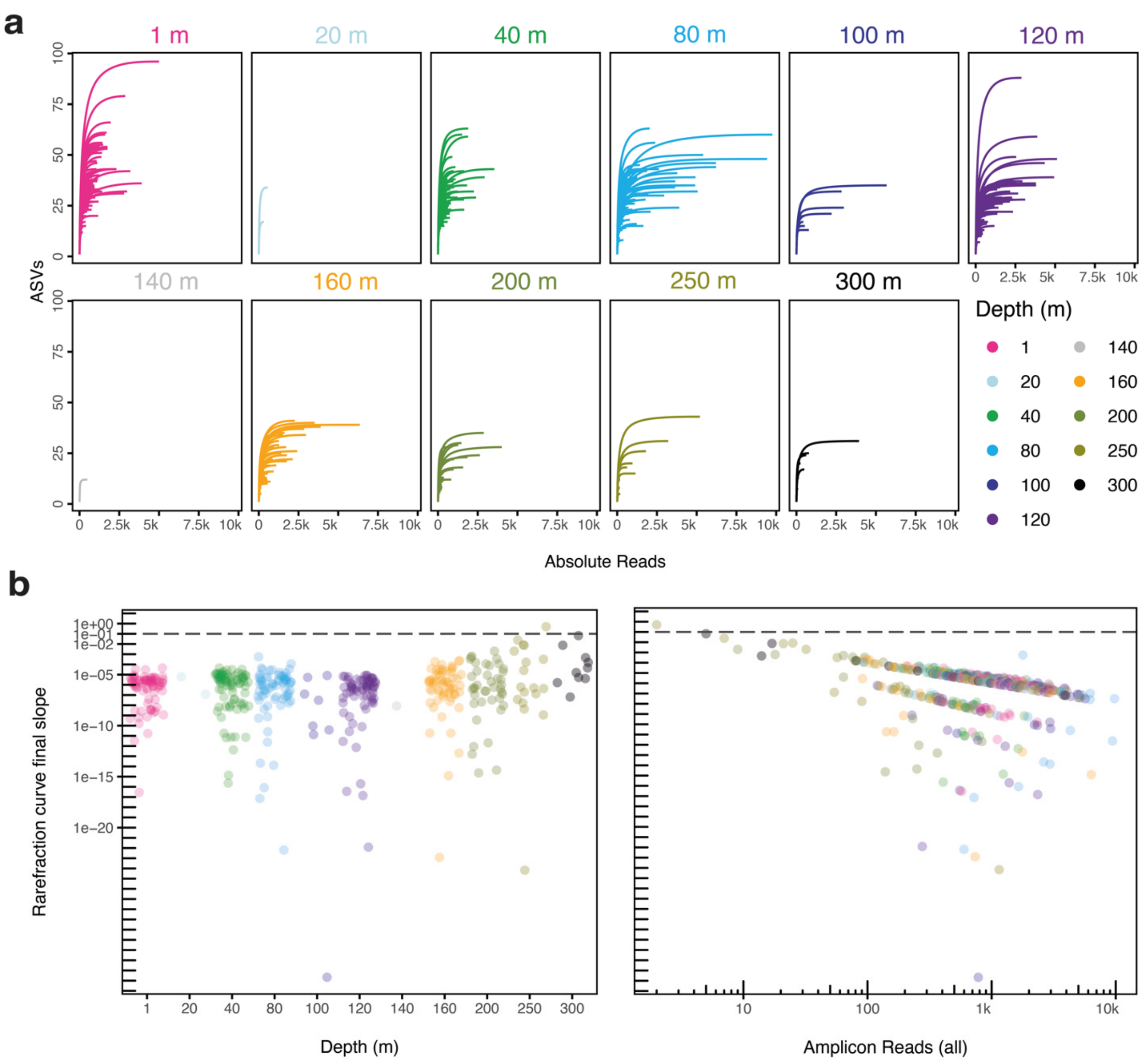


**Fig. S9. Rarefaction analysis of plastid amplicons only for the 431 samples used in the study** at all depths down to 300 m to determine sequencing saturation (samples from 1-140 m with <50 plastid amplicons were not considered). (a) Rarefaction curves with number of plastid ASVs vs. number of absolute reads. (b) The final slope of the rarefaction curves plotted vs. depth and number of plastid amplicons. Final slopes of below 0.1 are plotted below the dotted line and are considered to have reached saturation. Note that three samples from 250 to 300 m did not reach saturation.

**Supplementary References**

1. Simmons MP, Bachy C, Sudek S, van Baren MJ, Sudek L, Ares M, et al. Intron invasions trace algal speciation and reveal nearly identical Arctic and Antarctic *Micromonas* populations. *Mol Biol Evol* 2015; **32**: 2219–2235.
2. Choi CJ, Bachy C, Jaeger GS, Poirier C, Sudek L, Sarma VVSS, et al. Newly discovered deep-branching marine plastid lineages are numerically rare but globally distributed. *Current Biology* 2017; **27**: R15–R16.
