# Supplementary material for: Recurring seasonality exposes dominant species and niche partitioning strategies of open ocean picoeukaryotic algae
Source: Commun Earth Environ. 2024 May 20;5(1):266. doi: 10.1038/s43247-024-01395-7 (PMC11106004; doi:10.1038/s43247-024-01395-7)
Supplement: Supplementary file 3 — Description of Additional Supplementary Files [file 43247_2024_1395_MOESM3_ESM.pdf]

Description of Additional Supplementary Files

File name: Supplementary Data S1.

File Description: Table of collection data, oceanographic context (vertical zone 0=ML, 1=upper euphotic, 2=DCM, 3=winter mode layer, 4&5=ventilated thermocline), environmental data, and sequencing data for all 431 DNA samples used in the study.

File name: Supplementary Data S2

File Description: Averages of environmental data and percent prasinophyte of plastid amplicons by depth and stability period. '--' indicates insufficient data for a mean to be calculated.

File name: Supplementary Data S3.

File Description: Summary of parameters and results of statistical tests (Kruskal-Wallis Dunn, Spearman correlations, and ANOSIM).

File name: Supplementary Data S4.

File Description: Global distribution of the dominant persistent and other notable sub-species variants at BATS (with value represented as “Maximum percentage of plastids”).
