# Supplementary material for: Recurring seasonality exposes dominant species and niche partitioning strategies of open ocean picoeukaryotic algae
Source: Commun Earth Environ. 2024 May 20;5(1):266. doi: 10.1038/s43247-024-01395-7 (PMC11106004; doi:10.1038/s43247-024-01395-7)
Supplement: Supplementary file 8 — reporting summary [file 43247_2024_1395_MOESM8_ESM.pdf]

nature portfolio

Corresponding author(s): Alexandra Z. Worden

Last updated by author(s): YYYY-MM-DD

Reporting Summary

Nature Portfolio wishes to improve the reproducibility of the work that we publish. This form provides structure for consistency and transparency in reporting. For further information on Nature Portfolio policies, see our Editorial Policies and the Editorial Policy Checklist.

Statistics

For all statistical analyses, confirm that the following items are present in the figure legend, table legend, main text, or Methods section.

n/a	Confirmed	
☐	☒	The exact sample size (*n*) for each experimental group/condition, given as a discrete number and unit of measurement
☐	☒	A statement on whether measurements were taken from distinct samples or whether the same sample was measured repeatedly
☐	☒	The statistical test(s) used AND whether they are one- or two-sided<br>*Only common tests should be described solely by name; describe more complex techniques in the Methods section.*
☐	☒	A description of all covariates tested
☐	☒	A description of any assumptions or corrections, such as tests of normality and adjustment for multiple comparisons
☐	☒	A full description of the statistical parameters including central tendency (e.g. means) or other basic estimates (e.g. regression coefficient) AND variation (e.g. standard deviation) or associated estimates of uncertainty (e.g. confidence intervals)
☐	☒	For null hypothesis testing, the test statistic (e.g. *F*, *t*, *r*) with confidence intervals, effect sizes, degrees of freedom and *P* value noted<br>*Give P values as exact values whenever suitable.*
☒	☐	For Bayesian analysis, information on the choice of priors and Markov chain Monte Carlo settings
☒	☐	For hierarchical and complex designs, identification of the appropriate level for tests and full reporting of outcomes
☐	☒	Estimates of effect sizes (e.g. Cohen's *d*, Pearson's *r*), indicating how they were calculated

Our web collection on statistics for biologists contains articles on many of the points above.

Software and code

Policy information about availability of computer code

Data collection	No software was used
Data analysis	PhyloAssigner (source code at github https://github.com/BIOS-SCOPE/PhyloAssigner_python_UCSB), MAFFT (https://mafft.cbrc.jp/alignment/software/), MUST (https://doi.org/10.1093/nar/21.22.5264), jModeltest (10.1093/molbev/msn083), PhyML (https://doi.org/10.1093/sysbio/syq010), rstatix version 0.7.2 (https://cran.r-project.org/web/packages/rstatix/index.html), vegan 2.6.4 (https://cran.r-project.org/web/packages/vegan/vegan.pdf), tidyverse 2.0.0 (https://ggplot2.tidyverse.org/), gplots 3.1.3 (https://cran.r-project.org/web/packages/gplots/index.html), readxl 1.4.3 (https://readxl.tidyverse.org/), lubridate 1.9.3 (https://lubridate.tidyverse.org/), patchwork 1.2.0.9000 (https://patchwork.data-imaginist.com/)

For manuscripts utilizing custom algorithms or software that are central to the research but not yet described in published literature, software must be made available to editors and reviewers. We strongly encourage code deposition in a community repository (e.g. GitHub). See the Nature Portfolio guidelines for submitting code & software for further information.

Data

Policy information about availability of data

All manuscripts must include a data availability statement. This statement should provide the following information, where applicable:

- Accession codes, unique identifiers, or web links for publicly available datasets
- A description of any restrictions on data availability
- For clinical datasets or third party data, please ensure that the statement adheres to our policy

Oceanographic and environmental data (temperature, salinity, CTD-derived Chl fluorescence, Chl a, nitrate+nitrite, phosphate) can be found in the BCO-DMO repository (https://www.bco-dmo.org/dataset/861266 and http://lod.bco-dmo.org/id/dataset/3782) and at the BATS website (https://bats.bios.asu.edu/bats-data/) as well as in supplementary tables for data from a narrower set of information used in this manuscript. V1-V2 16S rRNA gene amplicons data can be found in the NCBI SRA repository under the project number PRJNA769790.

Human research participants

Policy information about studies involving human research participants and Sex and Gender in Research.

Reporting on sex and gender	N/A
Population characteristics	N/A
Recruitment	N/A
Ethics oversight	N/A

Note that full information on the approval of the study protocol must also be provided in the manuscript.

Field-specific reporting

Please select the one below that is the best fit for your research. If you are not sure, read the appropriate sections before making your selection.

☐ Life sciences ☐ Behavioural & social sciences ☒ Ecological, evolutionary & environmental sciences

For a reference copy of the document with all sections, see nature.com/documents/nr-reporting-summary-flat.pdf

Ecological, evolutionary & environmental sciences study design

All studies must disclose on these points even when the disclosure is negative.

Study description	We document vertical and seasonal patterns of a diverse suite of eukaryotic algae in the North Atlantic Subtropical Gyre using 16S rRNA V1-V2 amplicon sequence variants and contextual data. Sampling was performed monthly over four years at the Bermuda Atlantic Time-series Study at depths from 0- 300 m totalling 79 profiles and 431 samples.
Research sample	The main samples are seawater samples from which total DNA is extracted and algal groups determined based on 16S rRNA. These samples allow for a snapshot of the algal community (at a high taxonomic level) at that particular time and depth.
Sampling strategy	There was monthly sampling of the upper 300 m, with increased sampling over several days in the winter deep mixing period and the summer stratified period.
Data collection	Seawater samples for DNA were collected at 8 depths between 1 and 300 m using Niskin bottles affixed to a conductivity-temperature-depth (CTD) profiling rosette. Four L of seawater were filtered through 0.22 µm SterivexTM (Millipore) filters for DNA.
Timing and spatial scale	Data was collected approximately monthly from July 2016 to December 2019, with multi-day sampling in BIOS-SCOPE process cruises in September 2016, April 2017, July 2018, and July 2019. Fifty-six profiles were within 5 km of the BATS station (31°40' N, 64°10' 640 W), while the remaining 23 were within 6 to 109 km (Fig. 1a).
Data exclusions	Samples from 1-140 m with <50 plastid amplicons were not considered.
Reproducibility	This is an environmental study so not reproducible in the same was as a lab experiment. However, we do make sequences and data processing procedures publicly available.
Randomization	Samples were grouped according to water column depth zone (surface, mixed layer, deep chlorophyll maximum) and water column stability period (deep mixing, spring transition, summer stratified, and autumn transition) based on measured qualities of the water column.

Blinding	Blinding was not possible since it was important to record the context of the samples collected.

Did the study involve field work? ☒ Yes ☐ No

Field work, collection and transport

Field conditions	Fieldwork was performed onboard the RV Atlantic Explorer and RV Endeavor. Sea conditions were choppy during the winter deep mixing time, which precluded sampling from happening in March 2017.<br>Surface water temperatures ranged from 20.86+/-0.75 deg C (DM) to 21.09+/-0.67 deg C (ST) and reached a high of 27.33+/-2.02 deg C (SS) then decreased to 24.15+/-1.40 deg C (AT).
Location	Most sampling was within 5 km of the BATS station (31°40' N, 64°10' 640 W), while the remaining samples were collected within 6 to 109 km (Fig. 1a).
Access & import/export	All samples were collected in compliance with local and international guidelines; Export permits were used for shipment of seawater samples from Bermuda to the various analyzing labs.
Disturbance	N/A

Reporting for specific materials, systems and methods

We require information from authors about some types of materials, experimental systems and methods used in many studies. Here, indicate whether each material, system or method listed is relevant to your study. If you are not sure if a list item applies to your research, read the appropriate section before selecting a response.

Materials & experimental systems

n/a	Involved in the study
☒	☐ Antibodies
☒	☐ Eukaryotic cell lines
☒	☐ Palaeontology and archaeology
☒	☐ Animals and other organisms
☒	☐ Clinical data
☒	☐ Dual use research of concern

Methods

n/a	Involved in the study
☒	☐ ChIP-seq
☒	☐ Flow cytometry
☒	☐ MRI-based neuroimaging
